# Abnormal Reginal Homogeneity in Left Anterior Cingulum Cortex and Precentral Gyrus as a Potential Neuroimaging Biomarker for First-Episode Major Depressive Disorder

**DOI:** 10.3389/fpsyt.2022.924431

**Published:** 2022-06-01

**Authors:** Yan Song, Chunyan Huang, Yi Zhong, Xi Wang, Guangyuan Tao

**Affiliations:** ^1^Nanning Fifth People's Hospital, Nanning, China; ^2^Department of Cardiology, Tongren Hospital of Wuhan University (Wuhan Third Hospital), Wuhan, China; ^3^Peking University Sixth Hospital, Peking University Institute of Mental Health, NHC Key Laboratory of Mental Health (Peking University), Beijing, China; ^4^Department of Mental Health, Taihe Hospital, Hubei University of Medicine, Shiyan, China

**Keywords:** regional homogeneity, major depressive disorder, rs-fMRI, support vector machine, biomarker

## Abstract

**Objective:**

There is no objective method to diagnose major depressive disorder (MDD). This study explored the neuroimaging biomarkers using the support vector machine (SVM) method for the diagnosis of MDD.

**Methods:**

52 MDD patients and 45 healthy controls (HCs) were involved in resting-state functional magnetic resonance imaging (rs-fMRI) scanning. Imaging data were analyzed with the regional homogeneity (ReHo) and SVM methods.

**Results:**

Compared with HCs, MDD patients showed increased ReHo in the left anterior cingulum cortex (ACC) and decreased ReHo in the left precentral gyrus (PG). No correlations were detected between the ReHo values and the Hamilton Rating Scale for Depression (HRSD) scores. The SVM results showed a diagnostic accuracy of 98.96% (96/97). Increased ReHo in the left ACC, and decreased ReHo in the left PG were illustrated, along with a sensitivity of 98.07%(51/52) and a specificity of100% (45/45).

**Conclusion:**

Our results suggest that abnormal regional neural activity in the left ACC and PG may play a key role in the pathophysiological process of first-episode MDD. Moreover, the combination of ReHo values in the left ACC and precentral gyrusmay serve as a neuroimaging biomarker for first-episode MDD.

## Introduction

Major depressive disorder (MDD) is a very prevalent psychiatric disorder that significantly impacts patients' quality of life and physical health. According to the World Health Organization, more than 300 million people in the world suffered from depression. The suffering caused by self-mutilation, suicide, and other behaviors of depression patients to patients and their families and the loss to society cannot be ignored (1). In the past few decades, researchers have sought breakthroughs in the diagnosis of depression, from relying solely on symptomatic diagnosis to the birth of various markers, such as blood biomarkers and molecular genetics biomarkers: BDNF Val66Met, 5-HTTLPR risk gene biomarkers, and gut microbiota (2–4). However, a simple and objectively effective diagnostic marker has not been found.

Functional Magnetic Resonance Imaging (fMRI) is a feasible non-invasive medical imaging to examine the structure and connectivity of the brain (5). fMRI can be utilized to explore the working mechanism and regularity of the brain in the resting state, which mainly reflects the functional connection characteristics of the neural network of the brain (6). The principle of fMRI is to capture the hemodynamic changes under neural activity. Given that the brain's neural activity requires the local blood flow with sufficient oxygen supply, increasing the local blood oxygen supply results in the change in blood oxygen concentration, leading to the change of the magnetic resonance signal in the local brain area. This latter change can be detected by The Blood Oxygenation Level Dependent (BOLD) signal of each voxel point of the brain and recorded as the magnetic resonance imaging signal (7). Therefore, fMRI can decipher the differences of BOLD signals in various regions in the brain of depressed patients from healthy populations, facilitating the diagnosis of MDD.

A large number of studies have found that patients with depression have abnormal signals in various brain regions on fMRI (8–11). The most prominent regions are the medial prefrontal cortex, the limbic system, and the default network (12–15). Adolescent patients with MDD have been associated with heightened connectivity within default mode network (DMN) regions and diminished connectivity within FPN regions (16). A study of MDD patients with somatic symptoms showed that both ReHo and ALFF values in the bilateral precentral gyrus, postcentral gyrus, and left paracentral gyrus were lower than those in the control group (17). First-episode, treatment-naive patients with MDD showed decreased activity in the left dorsolateral prefrontal cortex and bilateral medial orbitofrontal cortex (18), and reduced ALFF was found in bilateral orbital frontal cortex (OFC), while increased ALFF in the bilateral temporal lobe extending to theinsular and left fusiform cortices in MDD patients compared to healthy controls (19). A meta-analysis of whole-brain rs-fMRI studies found that MDD patients displayed decreased ALFF in the bilateral cerebellum and bilateral precuneus cortex (20). The inconsistency of these results may be related to the interference of different disease courses, medications, and other factors. Therefore, selecting patients with first-episode drug-naïve depression as study subjects can reduce the interference of these confounding factors.

The support vector machine (SVM) has solved clinical problems since the mid-nineties. Several studies have used SVM combined with neuroimaging data to explore the diagnosis and treatment response prediction of depression at the individual level to improve the diagnostic accuracy of depression (21–27). By using SVM analysis, the possibility of distinguishing MDD from healthy controls by using the extracted abnormal ReHo values in brain regions can be examined in our study. We aimed to explore specific or distinctive alterations in first-episode MDD and whether the alterations could be used to separate first-episode MDD from healthy controls. We hypothesized that first-episode untreated depression patients have multiple abnormal ReHo brain regions, and these abnormal brain regions singly/combined as a biomarker to assist in diagnosing depression patients.

## Methods

### Participants

Fifty-two first-episode MDD patients and 45 age-, gender-, education-matched, health controls were recruited from the First Affiliated Hospital of Guangxi Medical University, China. All participants were right-handed. The patient's final diagnoses were independently confirmed by two experienced psychiatrists using the Structured Clinical Interview of the DSM-IV (SCID) (28) and assessed by the 17-item Hamilton Rating Scale for Depression (HRSD-17). The inclusion criteria for MDD patients were as follows: first major depressive episode; 17-item Hamilton Rating Scale for Depression (HRSD-17) total scores ≥17. Exclusion criteria were as follows for the patients: any history of head injury or lost consciousness, serious physical or neurological illness, other mental disorders meeting DSM-IV diagnostic criteria, such as a cute physical illness, substance abuse or dependence, schizophrenia, bipolar disorder. None of the healthy controls had a severe physical illness, history of mental disorders, or family history of mental disorders.

Each participant has submitted a written informed consent before enrollment. The study was approved by the Medical Research Ethics Committee of the First Affiliated Hospital of Guangxi Medical University, China, and performed in accordance with the Declaration of Helsinki.

### Image Acquisition

The resting-state MRI data were obtained by using an Achieva 3.0T scanner (Philips, Amsterdam, the Netherlands) at the First Affiliated Hospital of Guangxi Medical University in the first day after enrollment. All the participants were instructed to lie still, close their eyes, and remain awake during the scan. The resting-state functional images were employed, using an echo-planar imaging sequence with the following parameters: repetition time/echo time (TR/TE) 2000/30ms, 31 slices, 90° flip angles, 22 cm ×22 cmFOV, 5 mm slice thickness, and 1 mm gap.

### Data Preprocessing

DPARSF software in MATLAB was used to preprocess imaging data (29). Due to initial signal instability and participants' adaption time, the first five-time points were deleted in order to minimize the influence of participants' adaption time and the instability of the initial signal. Slice time and head motion were corrected.

All imaging data were with a maximum displacement in the x-, y-, or z-axis no more than 2 mm and maximum angular rotation no more than 2°. The corrected imaging data were spatially normalized to the standard Montreal Neurological Institute space and resampled to 1 mm ×1 mm ×1 mm. The obtained fMRI data were temporally band-pass filtered (0.01–0.08 Hz) and linearly detrended. Several spurious covariates were removed from the imaging data, such as the signal from the ventricular seed-based region of interest, the six-head motion parameters obtained by rigid body correction, and the white matter-centered region. The global signal was regressed out during the processing of the resting-state functional connectivity data.

ReHo analysis was performed using REST software. The formula used to calculate ReHo according to the previous study (30).

### Classification Analysis

Distributions of age, years of education, and voxel-based comparisons of whole-brain ReHo maps were compared by using two-sample *t*-tests. The gender ratio was compared by using Chi-square test. The resulting statistical maps were set at a threshold (*p* <0.01) for multiple comparisons (GRF corrected, voxel significance: *P* <0.01; clustering significance: *p* <0.01). Furthermore, linear correlations were calculated between abnormal ReHo values and psychological performances. The significance threshold was set at *p* <0.05.

SVM analysis was applied to examine the possibility of distinguishing MDD from healthy controls by using the extracted abnormal ReHo values in brain regions. The method of SVM was operated using the LIBSVM software package in MATLAB. The best parameters including C (penalty coefficient) and gamma value were selected. Through the LIBSVM tool, the grid of parameters were evaluated and all the parameter settings' accuracies were acquired. The highest cross-validation accuracy of the parameter was determined.

## Results

### Demographics Characteristics and Clinical Information

A total of 52 MDD patients and 45 healthy controls were involved in the study. No significant differences in age, gender, and year of education were observed between the two groups. Demographic information and clinical characteristics were shown in Table 1.

**Table 1:** Clinical information of the participants.

**Characteristics**	**Patients (*n* = 52)**	**Healthy controls (*n* = 45)**	***t*(or *χ2*)**	***p*-value**
Gender (male/female)	52 (15/37)	45 (12/33)	0.057	0.811^A^
Age (years)	25.77 ± 5.41	24.85 ± 4.17	0.923	0.358^B^
Years of education(years)	12.46 ± 2.63	12.40 ± 3.40	0.100	0.920^B^
Illness duration(months)	1.62 ± 1.07			
HRSD scores	22.37 ± 3.98			

*HRSD, Hamilton Rating Scale for Depression; ^A^The p-value was obtained by a Chi-square test; ^B^The p-value was obtained by two-sample t-tests*.

### ReHo: Patients vs. Controls

**Figure 1:**
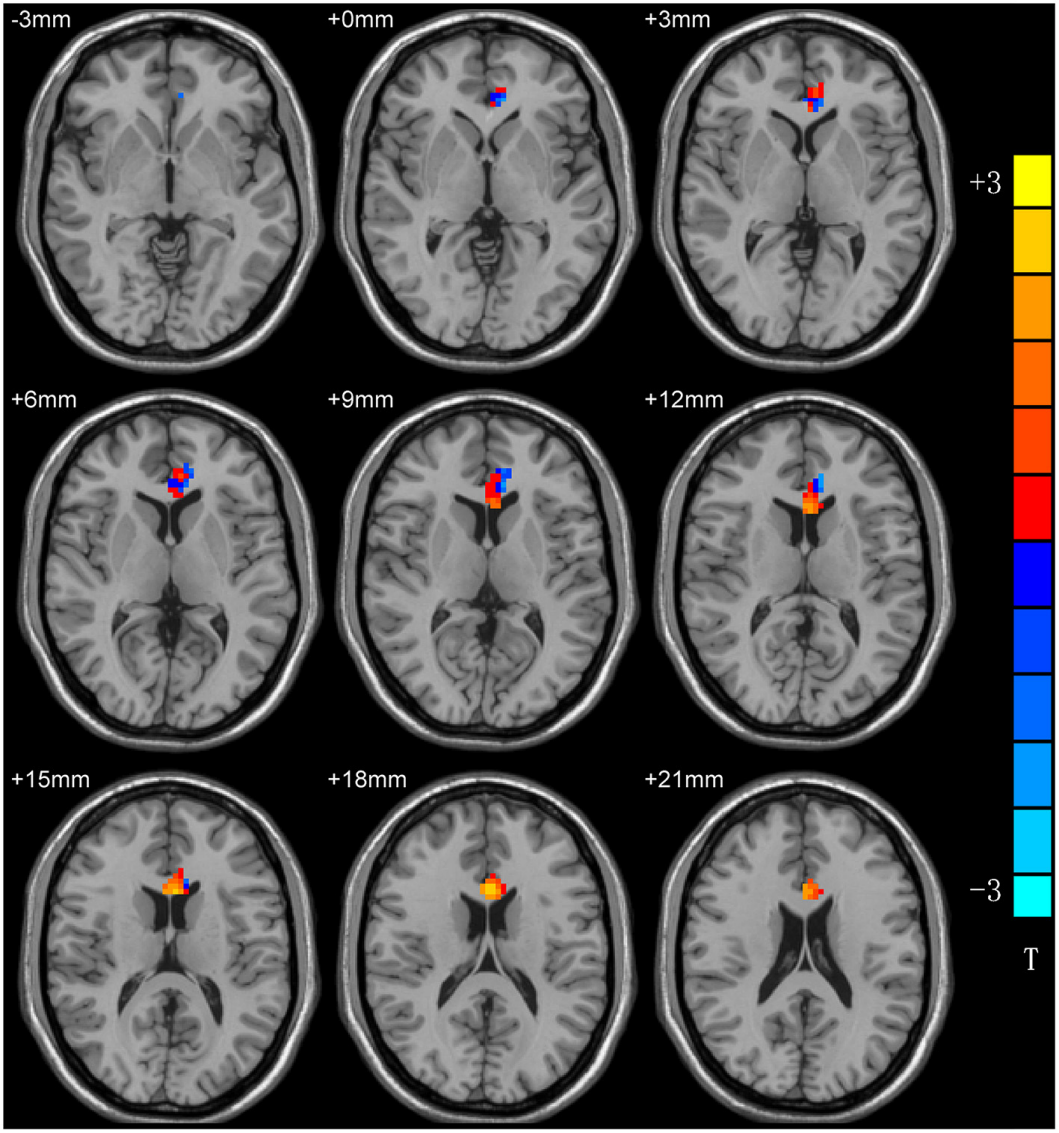
ReHo differences between patients with MDD and HCs. Red and blue denote higher and lower ReHo, respectivsely, and the color bars represent the *T*-values from the two-sample *t*-test of the group analysis. ReHo, regional homogeneity; MDD, major depressive disorder; HCs, healthy controls.

Compared with healthy controls, patients with MDD exhibited a significantly increased ReHo in the left anterior cingulum cortex (ACC) and decreased ReHo in the left precentral gyrus (PG) (Table 2; Figure 1).

**Table 2:** Brain regions with abnormal ReHo in MDD.

**Cluster location**	**Peak(MNI)**			**Number of voxels**	***t*-value**	***p*-value**
	**X**	**Y**	**Z**			
Patients>controls						
Left ACC	0	18	18	142	2.62	
Patients < controls						
Left PG	−30	−18	60	187	−3.45	

*ReHo, regional homogeneity; MDD, major depressive disorder; MNI, Montreal Neurological Institute; ACC, anterior cingulum cortex; PG, precentral gyrus*.

### The Correlations Between the ReHo Values and Other Factors

There was no correlations detected between the ReHo values and the Hamilton Rating Scale for Depression (HRSD) scores. There were no other factors such as gender and years of education in MDD patients were detected be related to the abnormal ReHo values.

### SVM Results

**Figure 2:**
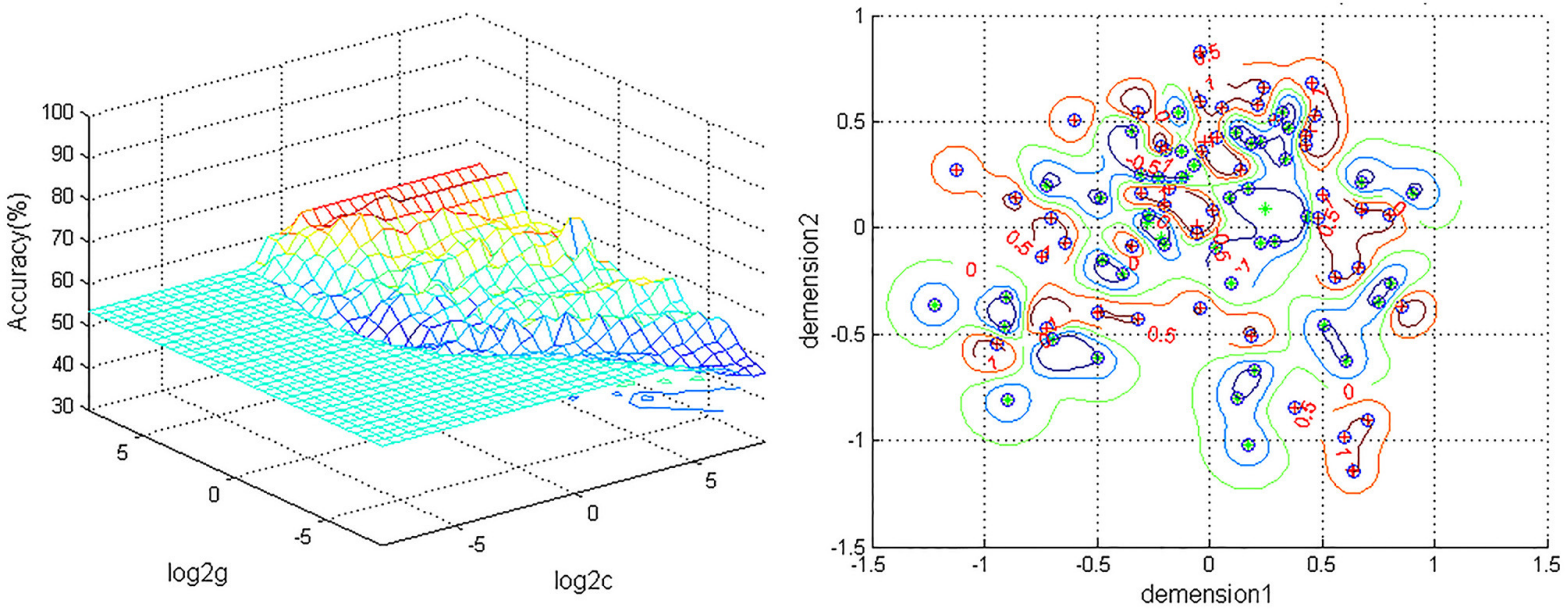
Depiction of classifications based on the SVM using a combination of ReHo values in the left ACC and PG to differentiate MDD patients from HCs. Left: SVM parameters result of 3D view. g, means gamma; c, penalty coefficient; Right, dimension 1 and dimension 2 represent the ReHo values in the left ACC and PG. Green crosses represent MDD patients, and the red crosses represent HCs. SVM, support vector machine; ReHo, regional homogeneity; ACC, anterior cingulum cortex; PG, precentral gyrus; MDD, major depressive disorder; HCs,s healthy controls.

A combination of the increased ReHo values in the left ACC and decreased ReHo in the left PG was used as a potential biomarker to diagnose MDD patients by the SVM method. The classification accuracies were as follows: diagnostic accuracy of 98.96% (96/97), a sensitivity of 98.07%(51/52), and a specificity of 100% (45/45) (Figure 2).

## Discussion

The objective, effective and rapid diagnosis of MDD has always been a hot spot in clinical research. However, there are no effective diagnostic methods for MDD, and its diagnosis still depends on depressive syndromes. Rs-fMRI research is increasingly used to assist clinical diagnosis. This study explored the utility of altered ReHo values in the left ACC and PG as a potential neuroimaging biomarker for the first-episode MDD by the SVM method.

The cingulum is a key component of the limbic lobe, and it also plays a key role in the DMN. The cingulum is the major interconnecting apparatus of all cerebral lobes (31). It has been described as the “seat of dynamic vigilance by which environmental experiences are endowed with an emotional awareness” by Papez (32). Based on the characteristics and anatomy of the cingulum gyrus, the cingulum gyrus is divided into four subregions such as the ACC, middle cingulum cortex, posterior cingulum cortex, and retrosplenial cortex (33). Anterior cerebrum The ACC is the first half of the cingulum gyrus, which is closely related to human cognitive execution, emotional processing, and other brain functions (34). When ACC is damaged, it will produce many clinical symptoms, including inattention, dysfunction of autonomic function regulation, emotional instability (35, 36). Studies have found that the ACC is prone to damage in depression (37, 38). Structural magnetic resonance study found that the anterior cingulum white matter fibrosis of the ACC recovered after 8 weeks of antidepressant treatment (39, 40). In the present study, similar results were noticed. Increased ReHo values of the left ACC and decreased PG were found in the patients with MDD, and thus we speculated that abnormal ReHo in the left ACC and PG has a critical role in the physiological processes of MDD. Another finding of this study was left-sided affected brain regions in MDD patients. As the ACC is considered to have a key role in the pathophysiology of the disorder, lack of normal symmetries in ACC has been long observed. In task-related fMRI, decreased functional connectivity between the left amygdala and the left ACC during negative stimuli in participants with MDD was found (41), while the increased depression duration was correlated with decreased perfusion of the right ACC (42). Consistent with the previous findings, our results suggest that lack of normal symmetries may be a characteristic for patients with MDD.

Prefrontal lobe dysfunction is associated with a variety of depressive symptoms, such as attention deficit, psychomotor retardation, executive dysfunction, etc., and is related to the treatment of depression (43). The prefrontal-striatal neural circuit underlies behavioral disinhibition (32). Previous studies have found that dorsal medial prefrontal gyrus lesions are associated with susceptibility to depression (44). In MDD patients, behavioral disinhibition is associated with increased suicidal behavior, mental agitation, impulsivity loss, and substance use disorders. related to (44, 45). Furthermore, Structural magnetic resonance studies found that the precentral gyrus volume was reduced in patients with depression compared with normal people (46). Carlson et al. also found that in patients with MDD, increased depression was associated with a decrease in PFC volume (47). A recent study also found that compared with MDD patients who did not attempt suicide, suicide attempters had a greater surface area in the left retrocentral gyrus and lateral occipital gyrus but a smaller surface area in the left superior frontal gyrus (48). These studies suggest that the damage to the precentral cortex in patients with depression is involved in the pathological mechanism of depression.

Antidepressants may have an effect on brain structure and function (49–51). Therefore, it is essential to select drug-naive patients as a starting point to minimize the potentialeffects of medication. Long illness duration may have a neurotoxic effect on brain structure (52). Guo and his companies found that the combination of abnormal ReHo in the right fusiform gyrus/cerebellar and right superior/middle occipital gyrus showed an accuracy of 83.05%, the sensitivity of 90.32%, and specificity was 75.00%, which was used to distinguish depressive MDD patients from non-depressed MDD patients, and the combination of abnormal ReHo in right fusiform gyrus/cerebellar and left precentral gyrus showed the accuracy of 98.41%, sensitivity of 96.77%, and specificity of 100.00%, used to distinguish depressive MDD patients from healthy controls (21). In the present study, our study found abnormal ReHo values in ACC and PG within patients with first-episode MDD. Furthermore, an SVM was used to combine the ReHo signals of these two abnormal brain regions as a biomarker for diagnosing MDD with an accuracy of 98.96%, a sensitivity of 98.07%,and a specificity of 100%. A slightly larger sample size, unmedicated, and shorter disease duration may explain our results may have better clinical value.

Some limitations exist in the study. First, the age of the patients is concentrated in the young and the mean illness duration is <2 months. Further study is needed to justify these results in order to enhance the possibility of generalizing the results of this study to MDD patients with various clinical characteristics. Second, we did not know whether changes in the left anterior Cingulum cortex and Precentral gyrus occurred before or as a result of MDD. A long-term follow-up observation may help us to understand the cause and effect.

## Conclusion

In conclusion, the altered ReHo in the left ACC and PG may be state-related changes of MDD. Also, the combination of increased ReHo in the left ACC and left PG may be a potential neuroimaging biomarker for the first-episode MDD.

## Data Availability Statement

The original contributions presented in the study are included in the article/supplementary material, further inquiries can be directed to the corresponding author/s.

## Ethics Statement

The studies involving human participants were reviewed and approved by the Medical Research Ethics Committee of the First Affiliated Hospital of Guangxi Medical University, China. The patients/participants provided their written informed consent to participate in this study.

## Author Contributions

XW and GT contributed to the conception and design of the study. CH and YZ supervised the progress of the study. YS performed the data analysis and wrote the manuscript. All authors contributed to manuscript revision, and approved it for publication.

## Conflict of Interest

The authors declare that the research was conducted in the absence of any commercial or financial relationships that could be construed as a potential conflict of interest.

## Publisher's Note

All claims expressed in this article are solely those of the authors and do not necessarily represent those of their affiliated organizations, or those of the publisher, the editors and the reviewers. Any product that may be evaluated in this article, or claim that may be made by its manufacturer, is not guaranteed or endorsed by the publisher.
